# Prevalence of HCV among the high risk groups in Khyber Pakhtunkhwa

**DOI:** 10.1186/1743-422X-8-296

**Published:** 2011-06-11

**Authors:** Ijaz Ali, Lubna Siddique, Latif U Rehman, Najib U Khan, Aqib Iqbal, Iqbal Munir, Farzana Rashid, Sana U Khan, Safira Attache, Zahoor A Swati, Mehwish S Aslam

**Affiliations:** 1 Institute of Biotechnology and Genetic Engineering, Khyber Pakhtunkhwa Agricultural University, Peshawar, Pakistan; 2 Lahore College for Women University, Lahore, Pakistan; 3 Kohat University of Science and Technology, Kohat, Pakistan

## Abstract

Hepatitis C is an infectious disease, caused by blood borne pathogen; the Hepatitis C Virus. In this study we analyzed blood samples collected from various risk groups for the prevalence of anti-HCV and active HCV infection with the help of Immunochromtographic tests and nested PCR. The prevalence of active HCV infection among the high risk groups was 15.57% (26/167). The prevalence of HCV in individual risk groups was 15%, 28%, 8%, 14.28% and 14.28% in the case of thalassemics, dialysis, major surgery group, dental surgery group and injection drug users respectively. Our analysis reveals the fact that health care facilities in the Khyber Pakhtunkhwa province of Pakistan are contributing a great deal towards the spread of HCV infection.

## Introduction

Hepatitis C is an infectious disease affecting the liver, caused by the hepatitis C virus (HCV). HCV, a member of the *Flaviviridae *family, was discovered as a new viral agent causing non-A, non-B hepatitis by Choo and co-workers in 1989 [1]. WHO estimated the global prevalence of Hepatitis C as 3% [2]. In developing countries where resources and facilities may be significantly limited, the prevalence of HCV is higher as compared to the developed world [3].

HCV is transmitted through contaminated blood transfusion, surgery, surgical instruments, dental surgery and excessive dental consultations, sexual contacts, drug abuses, sharing of the house hold items such as razors, toothbrushes and shaving from the barber [4-6]. Some health care procedures, i.e., surgical and dental treatments, have recently been indicated as risk factors for acute HCV [7]. In Pakistan, blood transfusion is still a major source of HCV transmission. Possible reasons for this include lack of resources, weak infrastructure, ill-equipped resources, poorly trained staff, inadequate policy implementation, frequent power breakdown and ineffective screening of blood donors for anti-HCV antibody [8]. Regular blood transfusion in patients with hereditary hemolytic anemia, particularly Thalassemia, has improved their overall survival, but carries a definite risk of acquisition of blood-borne virus infections, especially viral hepatitis [9].

Major risk factors associated with the transmission of HCV were never investigated at molecular level in Khyber Pakhtunkhwa. In this study, we have analyzed patients belonging to various risk groups for the prevalence of anti HCV and active HCV infection. The risk groups included thalasemics, major surgery group, dental surgery group, dialysis group and IDUs (Injection drug users).

## Methods and materials

### Sampling

After having approved the study by the ethics committee of the Institute of Biotechnology and Genetic Engineering, Peshawar, a total of 167 blood samples were collected from patients belonging to various risk groups including thalassemics, major surgery group, dental surgery group, injection drug users and dialysis group. Each individual duly signed a proforma containing information about his/her previous exposure to a risk factor, age, sex etc. 5 mL of blood sample was collected in EDTA-tubes in each case and immediately transported to IBGE for serum isolation. Sera were stored at -20 C until used. All experiments were performed inn accordance with the ethical standards of the Declaration of Helsinki.

### Immunochromatographic test (ICT)

Screening for HCV positive samples was carried out with the help of Immunochromatographic tests. Strips used were from accurate and Acon (Acon, USA) according to the manufacturers instructions. Samples positive by ICT were further processed for next step evaluation.

### RNA Extraction and RT-PCR

HCV RNA was extracted from 100 μl serum by using Anagen RNA extraction kit (Purescript, USA) according to the manufacturer's instructions. Qualitative detection of serum HCV RNA was performed by Reverse transcription PCR as mentioned previously [10].

### Gel electrophoresis

PCR products were analyzed on 2% agarose gel prepared in 0.5% TBE buffer, stained with Ethedium bromide (10 μ g/ml) as florescent dye. A 100-bp DNA ladder (Gibco BRL) was used as DNA size marker. Gels were photographed using Alpha quant (Alpha Innotech).

The data was analyzed with SPSS version 10.0 for windows or Microsoft Excel. Frequencies of hepatitis C in different risk groups were calculated in percentages.

## Results

A total of 167 blood samples were screened including thalassemic patients, dialysis patients, people having major surgeries, people having undergone dental surgeries (minor and major) and injection drug users.

Initial screening was done for anti-HCV using ICT strips from two different sources. Confirmation of active HCV infection was carried out with the help of RT-PCR. Out of 167 samples, 26 (15.57%) were HCV positive both for anti-HCV and HCV RNA. Prevalence of active HCV infection in individual risk groups was 15%, 28%, 8%, 14.28% and 14.28% in thalasemics, dialysis, major surgery group, dental surgery group and injection drug users respectively (Table 1).

**Table 1:** Prevalence of HCV in individual risk groups

S.No	Major Risk groups (N)	ICT positive	PCR positive
1	Thalassemia (40)	6 (15%)	6 (15%)

2	Dialysis (25)	7 (28%)	7 (28%)

3	Major surgery (25)	2 (8%)	2 (8%)

4	Dental surgery (35)	5 (14.28%)	5 (14.28%)

5	IDUs (42)	6 (14.28%)	6 (14.28%)

Total	167	26 (15.56%)	26 (15.56%)

**Figure 1:**
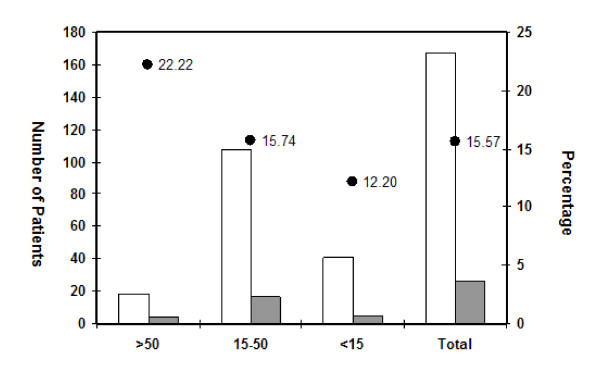
**Age wise distribution of all the patients belonging to various risk groups**. White bars represent total number of patients in each age group, grey bars represent HCV positive patients and filled circles show the percentage of HCV infected individuals.

Exposure to various HCV risk factors is preferentially gender specific in our country mainly due to our social set up. Exposure of females to some of the risk factors is limited. Among the observed population, 104 (62.27%) were male and 63 (37.72%) were female out of which 18 (17.30%) males and 8 (12.68%) females were positive for anti-HCV as well as HCV RNA (Table 2). In terms of age distribution, the subjects were grouped into three categories Lowest prevalence (12.20%) was recorded in the case of patients with age 15 years or below, while in the case of older age group (50 years and above), the highest HCV prevalence (22.22%) was recorded (Figure 1).

**Table 2:** Sex wise prevalence of active HCV infection

Sex	Total samples	Positive cases	Negative cases	Prevalence (%)
Male	104	18	86	17.30%

Female	63	8	55	12.68%

Total	167	26	141	15.57%

Out of the total 167 samples from different risk groups included in the study, 3.59% were positive in the case of thalassemia, 4.19% in dialysis, 1.19% in major surgery, 2.99% in dental surgery and 3.59% in injection drug users. Highest prevalence was observed in dialysis patients (Table 3). Major risk factors for HCV transmission in KPK turned out to be dialysis followed by Thalasemia, Injection drug use and dental surgery.

**Table 3:** Prevalence of HCV among the high risk groups

Sex	Total samples	HCV positive	Thalassemia	Dialysis	Major surgery	Dental surgery	IDUs
Male	104	18		5	Nil	2	6

Female	63	8	1	2	2	3	Nil

Total	167	26	06	07	02	05	06

		15.56%	3.59%	4.19%	1.19%	2.99%	3.59%

## Discussion

Thalassemia major patients are among the high risk groups for HCV infection. Earlier studies from various regions of Pakistan have reported high prevalence of HCV (20.5%-60%) among thalasemics [11-14] In this study The prevalence of anti-HCV or HCV RNA in individual group was 15% while among the high risk groups, it was 3.59% (Table 1, 3) which is lower as compared to the previous estimates [15]. Majority of the studies undertaken in Pakistan have relied on anti-HCV detection in thalasemics using ICT devices and active infection was never investigated. The limitations of ICT devices for the detection of anti-HCV have been documented earlier [16]. Our own observation also re-enforces the same [Unpublished data]. In KPK, where resources are extremely limited to screen anti-HCV, contaminated blood transfusion seems to be the most important factor contributing a great deal towards the spread of HCV in Thalasemic patients.

Major surgery also contributes towards HCV transmission. In Khyber Pakhtunkhwa province of Pakistan, the public sector hospitals are not adequately equipped for screening of blood and blood products and according to our own observation proper sterilization procedures are also not practiced due to various reasons including burden of patients undergoing surgeries or sometimes lack of awareness about the transmission of HCV. Earlier studies reported the prevalence of HCV in major surgery groups as, 6.92%, 16.6%, 11.66% and 11.26% [17-20]. In this study, 2/25 (8%) of the individuals with no history of HCV infection prior to major surgery turned out to be positive for anti-HCV and HCV RNA (Table 1). Though the prevalence of active HCV infection among the major surgery group is less than the previous reports, however it is still alarmingly high as compared to other parts of the world.

Dental surgery is one of the major risk factors for HCV transmission in Pakistan. Some studies have reported that dental procedures were the major source of exposure (39.7%) followed by injections (16.6%) and surgical procedures (16.6%) [21]. Also the contaminated dentist equipments were the source of HCV infection in 17.94% people [22]. In this study the prevalence of anti-HCV and active HCV infection in the individual dental surgery group was 14.28%. Detection of anti-HCV antibodies and HCV RNA in patients who have a recent history of dental surgery (major/minor) with no HCV infection prior to the surgery indicates that dental surgical and scaling instruments are not properly sterilized in our hospitals and clinics. Apart from the public sector hospitals, we observed that due to poor economic condition, many people consult dental quakes practicing in various parts of the province. None of the quakes were informed about the risk of HCV transmission as a result of contaminated equipments and they also were not aware about the sterilization procedures.

Hemodialysis is considered to be one of the major risk factors for HCV transmission. Some earlier studies reported that the prevalence of HCV in hemodialysis patients was 68% in Pakistan, 23.7% in Quetta and 24.7% in Lahore [23-25]). In India the prevalence of anti HCV is recorded as high as 83% in hemodialysis patients [26]. In this study the prevalence of anti-HCV antibodies and HCV RNA to be the highest as it was 28% in the individual risk group and 4.19% among the high risk groups (Table 3). The highest prevalence of HCV in dialysis group indicates the limitations of the screening procedures used in these units for the detection of anti-HCV.

It is evident from the previous studies conducted in Pakistan that injection drug use is a predominanat mode of HCV transmission [27,28]. 60% of the hepatitis C transmission in the United States is attributed to injection drug use [29] and 52% in Tehran [30]. In this study, prevalence of anti-HCV and HCV RNA among the IDUs was 14.28% in the individual group of IDUs while it was 3.59% among the entire load of samples tested. None of the IDUs had a previous history of blood transfusion or surgeries but all of them did share needles in the past. Although, awareness about needle sharing has increased in recent times, yet the poorly educated IDUs of KPK did not seem to be aware of the risk of needle sharing.

It is also noted in this study that the prevalence of HCV was higher in males (17.30%) as compared to females (12.68%) which is in conformity with another local study [18]. Higher prevalence of HCV in males is probably due to exposure to numerous risk factors. According to our cultural environment, females are only negligibly exposed to some of the risk factors e.g. tattooing, injection drug use, barbers etc. Higher prevalence in males seems to be associated with our cultural attributes.

## Conclusion

Lack of proper blood screening facilities in Khyber Pakhtunkhwa province and the lack of awareness about the possible transmission routes of HCV are contributing a great deal towards the spread of the infection among the population. Proper sterilization and screening procedures must be made mandatory on public sector health care units so as to avoid a far bigger threat of more HCV infections in the near future. The policy makers should formulate laws and ensure its implementation with respect to banning the unqualified dental quakes working in various parts of the province.

## Competing interests

The authors declare that they have no competing interests.

## Authors' contributions

IA designed the study and advised about the protocols. LS, LR, AI and SA carried out sampling, experimental procedures and manuscript preparation. MSA helped LS with lab work plus manuscript preparation. MSA, SK, IM, FR and ZAS critically reviewed and approved the manuscript. All authors read and approved the final manuscript.
